# Evaluation of the relationship between migraine and psoriasis: a case-control study^☆^

**DOI:** 10.1016/j.abd.2022.04.009

**Published:** 2023-01-19

**Authors:** Mohamad Sarkhani, Majid Rostami Mogaddam, Ghasem Fattahzadeh-Ardalani, Nasrin Fouladi

**Affiliations:** a Students Research Committee, School of Medicine, Ardabil University of Medical Sciences, Ardabil, Iran; b Department of Dermatology, School of Medicine, Ardabil University of Medical Sciences, Ardabil, Iran; c Department of Neurology, School of Medicine, Ardabil University of Medical Sciences, Ardabil, Iran; d Department of Community Medicine, School of Medicine, Ardabil University of Medical Sciences, Ardabil, Iran

**Keywords:** Psoriasis, Headache, Migraine disorders

## Abstract

**Background:**

Although several recent studies have attempted to describe the association between psoriasis and migraine, there is little data in this regard.

**Objective:**

To explore the relationship between migraine and psoriasis.

**Methods:**

A total of 312 patients with psoriasis and 312 age- and gender-matched controls without psoriasis were recruited in this case-control study. Based on the diagnosis of migraine, they were divided into 4 subgroups: psoriasis with (PM+) and without (PM-) migraine, and control with (CM+) and without migraine (CM-). The subgroups were compared regarding the migraine and psoriasis characteristics.

**Results:**

The mean (SD) age of patients and controls (139 males, in each group) was 43.2 (13.2) years. Psoriasis patients were significantly more likely to have migraine (OR = 2.789). Migraine with aura was significantly higher in the PM + group than in the CM + group (p = 0.007). The mean PASI score (p = 0.001), frequency of moderate and severe psoriasis (p = 0.048), and frequency of patients with PsA (p < 0.001) were significantly higher in PM + compared to PM-. The risk of migraine substantially increased with increasing psoriasis severity (OR = 2.062, OR = 3.248, and OR = 4.586 for mild, moderate, and severe, respectively), and with the presence of PsA (OR = 2.438 and OR = 12.930 for patients without and with PsA, respectively).

**Study limitations:**

Observational nature, not including all confounding factors, not addressing a cause-and-effect relationship.

**Conclusions:**

In comparison with the non-psoriatic control group, psoriasis patients are predisposed to a significantly higher risk of migraine, particularly migraine with aura, psoriasis patients with more severe disease and those with PsA have a markedly higher risk of having migraine, and the migraine headache index is significantly higher in psoriasis patients.

## Introduction

Psoriasis is a chronic and relapsing skin disease characterized by scaling skin plaques^1^ Psoriasis prevalence differs by age, gender, region, and ethnicity and is estimated to affect nearly 2%‒12% of the human population in various communities^2^ There is growing knowledge that psoriasis is beyond some “skin-only disease”. Psoriasis has been shown to have common systemic manifestations and significant comorbidities with several chronic inflammatory diseases, including rheumatoid arthritis, cardiovascular diseases, hypertension, diabetes, obesity, and hyperlipidemia.^3^

Migraine is a neurological disorder accompanied by inflammation. It is a prevalent disease, affecting almost 10%‒20% of the adult population worldwide.^4^ Migraine headaches are considered one of the primary causes of disability in daily activities. Migraines are ranked as the sixth reason for lost years of disability worldwide.^5^ Migraines are reported to have comorbidity with other inflammatory diseases and conditions, including rheumatoid arthritis,^6^ cardiovascular disease,^7^ obesity,^8^ and inflammatory bowel disease.^9^

The pathogenesis of migraine and psoriasis is not fully understood. However, exploring the proposed pathogenesis of these conditions suggests some shared overlaps and common pathophysiology between them. For instance, the role of endothelin and calcitonin gene-related peptide (CGRP) in migraine^10^, ^11^ and psoriasis pathogenesis^12^, ^13^ has been proposed. Nitric oxide has been implicated in migraine attacks,^14^ and its possible role in the pathogenesis and severity of psoriasis also has been suggested.^15^ Furthermore, psoriasis and migraine are significantly associated with shared comorbidities, including cardiovascular diseases and stroke.^3^, ^7^ Moreover, some of the shared biomarkers between psoriasis and migraine, namely cytokines, TNF-α, and interleukins, have been proposed as diagnostic biomarkers for psoriasis and migraine or their severity.^16^, ^17^

Given all the evidence, these two diseases seem to be inflammatory conditions with possible interrelation. Understanding this potential relationship is noteworthy and can provide new insight into the underlying mechanisms of both diseases. Some studies have concentrated on exploring the association between migraine and psoriasis. For example, two cohort studies have shown the increased risk of migraine in patients with psoriasis.^18^, ^19^ However, the migraine type was not addressed in these retrospective studies. A significant direct relationship between migraine and psoriasis was found in two cross-sectional studies,^20^, ^21^ while another study showed no association between migraine and psoriasis.^22^ However, none of the three studies examined the characteristics of migraine and psoriasis. One study revealed a higher frequency of migraine with aura in psoriatic patients with migraine than in migraine patients without psoriasis.^23^ However, the control group in this study was only selected from migraine cases, and consequently, the overall frequency of migraine in the psoriasis group was not evaluated compared to the control group or odds ratios. A recent study exploring the relationship between migraine and psoriasis characteristics found that psoriasis severity in the subgroup of psoriasis with migraine was higher than the subgroup of psoriasis without migraine.^24^ However, this study did not include a control group without psoriasis.

Given the inadequacy of the available information on the relationship between migraine and psoriasis, the present study aimed to investigate further the relationship between these two diseases through a case-control study.

## Methods

The present study was a case-control study. The number of cases and controls required for the study was determined based on the findings of a pilot study and using a sample size formula for matched case-control studies.^25^, ^26^ The calculations related to sample size were performed in StatsDirect Software (version 3.3.5, 2021, StatsDirect Ltd., UK) which uses the mentioned formula. The pilot study included 60 patients with psoriasis and 60 age- and gender-matched controls, which achieved the following results: correlation coefficient for exposure between cases and controls = 0.129, probability of exposure among the control group = 0.133; and odds ratio = 1.98. With these data and considering alpha = 0.05, power = 0.85, and controls per case ratio = 1, the sample size in each group was calculated as 312.

The psoriatic subjects were selected from patients referred to the dermatology clinic of a teaching hospital using a convenient sampling method. The inclusion criteria for the psoriasis group were diagnosis of psoriasis based on clinical examination by a dermatologist, age above 18 years, a psoriasis duration of at least six months, no other skin diseases, and consent for participation in the study. The control group was recruited from healthy people referred to the hospital clinics as the patients' companions and was matched with the psoriasis group in terms of age and gender. The control group met the following inclusion criteria: age above 18 years, without psoriatic disease, and consent to participate in the study. Exclusion criteria in both groups were as follows: use of psychiatric medications (like tranquilizers and anti-depressive agents), smoking, substance or alcohol addiction, presence of a systemic disease (including hypertension, diabetes, cardiac disease, renal dysfunction), neurological problems (including convulsions or congenital nervous system disorders), and non-migraine headaches.

Information regarding age, gender, and body mass index (BMI) was determined for all participants and recorded in a checklist for each individual. Psoriasis-related information, including type, severity, duration, affected body areas, and the existence of Psoriatic Arthritis (PsA), was collected for patients in the case group. The psoriasis severity was determined based on the PASI score,^27^ and PsA was diagnosed by PASCAR criteria.^28^ Then, psoriatic patients and the control group were assessed by a neurologist for migraine diagnosis and its characteristics, including migraine type, headache frequency (number of headache attacks in a month), headache severity (sum of the severity of the headache attacks in a month/number of attacks), headache duration (sum of the hours of headache attacks in a month/number of attacks), and migraine headache index (headache severity × headache duration × headache frequency/30)^29^ Migraine diagnosis was made according to the International Headache Society criteria^30^ and headache severity was evaluated by visual analog scale (VAS). The psoriasis group (Group P) and control group (Group C) were assigned into subgroups of psoriasis with migraine (PM+), psoriasis without migraine (PM-), control with migraine (CM+), and control without migraine (CM-) and compared in terms of migraine and psoriasis characteristics.

The Chi-Square test was applied to evaluate the migraine frequency between groups P and C. Given the non-normal distribution of quantitative variables, which was assessed by the Kolmogorov-Smirnov test, the quantitative variables were compared between two groups using the Mann-Whitney *U* test and among three or more groups using the Kruskal-Wallis test. The odds ratios were determined using the logistic regression analysis. The significance level was considered 0.05 for all analyses.

StatsDirect Software (version 3.3.5, 2021, StatsDirect Ltd., UK) was used to calculate the sample size. All other analyses were performed with SPSS for Windows (Version 25.0, IBM Inc., USA).

The present study received approval from the Research Ethics Committee at the Ardabil University of Medical Sciences on Nov. 16, 2020 (ethical code: IR.ARUMS.REC.1399.448). Participation in this study was voluntary, and all participants entered the study with informed consent.

## Results

The mean (SD) age of patients and controls (139 male, 173 females in each group) were 43.2 (13.2) years. The two groups had no significant difference in BMI (p = 0.079). The type of psoriasis was vulgaris in all patients. Psoriasis duration in most patients was 2 to 5 years (39.7%). The involved areas included lower limbs in 66.3%, upper limbs in 59.9%, neck, and head in 50.3%, and trunk in 41% of patients. Psoriasis severity was mild in 56.1%, moderate in 23.1%, and severe in 20.8%. Psoriatic arthritis was present in 5.8% of patients (Table 1).

**Table 1:** Basic information and migraine frequency in study participants.

Variable	Group		p-value
	Psoriasis	Control	
Gender			
Female	173 (55.4%)	173 (55.4%)	1.0
Male	139 (44.6%)	139 (44.6%)	
Age (years)	43.2 ± 13.2	43.2 ± 13.2	1.0
BMI			
Normal	144 (46.2%)	172 (55.1%)	0.079
Overweight	103 (33.0%)	84 (26.9%)	
Obese	65 (20.8%)	56 (17.9%)	
Duration			
< 2 years	63 (20.2%)	‒	‒
2‒5 years	124 (39.7%)	‒	
5‒10 years	87 (27.9%)	‒	
> 10 years	38 (12.2%)	‒	
Lower-limb involvement		‒	
No	105 (33.7%)	‒	‒
Yes	207 (66.3%)		
Upper-limb involvement		‒	
No	125 (40.1%)	‒	‒
Yes	187 (59.9%)		
Head & neck involvement		‒	
No	155 (49.7%)	‒	‒
Yes	157 (50.3%)		
Trunk involvement		‒	
No	184 (59.0%)	‒	‒
Yes	128 (41.0%)		
PASI score	7.7 ± 6.0	‒	‒
Psoriasis severity		‒	
Mild	175 (56.1%)	‒	‒
Moderate	72 (23.1%)	‒	
Severe	65 (20.8%)		
PsA		‒	
No	294 (94.2%)	‒	‒
Yes	18 (5.8%)		
Migraine			
No	246 (78.8%)	285 (91.3%)	p < 0.001
Yes	66 (21.2%)	27 (8.7%)	

The migraine frequency in the psoriasis group (21.2%) was significantly higher than in the control group (8.7%) (p < 0.001) (Table 1).

Migraine with aura and migraine headache index were significantly higher in the PM + group than in the CM + group (p = 0.007 and p = 0.015, respectively). However, there was no significant difference between these two groups in terms of headache severity (p = 0.422), frequency (p = 0.070), and duration (p = 0.247) (Table 2).

**Table 2:** The association between migraine characteristics and psoriasis.

Variable	Group		p-value
	PM+	CM+	
Migraine type			
Without aura	31 (47.0%)	21 (77.8%)	0.007
With aura	35 (53.0%)	6 (22.2%)	
Headache severity	7.6 ± 1.7	7.2 ± 1.9	0.422
Headache frequency	8.3 ± 1.8	7.5 ± 1.6	0.070
Headache duration	15.1 ± 6.8	13.0 ± 4.7	0.247
Migraine headache index	30.7 ± 16.5	23.0 ± 11.0	0.015

The mean PASI score (p = 0.001), frequency of moderate and severe psoriasis (p = 0.048), and frequency of patients with PsA (p < 0.001) were significantly higher in PM + compared to PM- (Table 3).

**Table 3:** The association between psoriasis characteristics and migraine.

Psoriasis characteristics		Group		p-value
		PM+	PM-	
Duration	< 2 years	13 (19.7%)	50 (20.3%)	0.830
	2‒5 years	24 (36.4%)	100 (40.7%)	
	5‒10 years	19 (28.8%)	68 (27.6%)	
	> 10 years	10 (15.2%)	28 (11.4%)	
Lower-limb involvement	No	16 (24.2%)	89 (36.2%)	0.068
	Yes	50 (75.8%)	157 (63.8%)	
Upper-limb involvement	No	24 (36.4%)	101 (41.1%)	0.490
	Yes	42 (63.6%)	145 (58.9%)	
Head & neck involvement	No	29 (43.9%)	126 (51.2%)	0.294
	Yes	37 (56.1%)	120 (48.8%)	
Trunk involvement	No	33 (50.0%)	151 (61.4%)	0.095
	Yes	33 (50.0%)	95 (38.6%)	
PASI score		10.4 ± 7.7	7.0 ± 5.2	0.001
Psoriasis severity	Mild	29 (43.9%)	146 (59.3%)	0.048
	Moderate	17 (25.8%)	55 (22.4%)	
	Severe	20 (30.3%)	45 (18.3%)	
PsA	No	56 (84.8%)	238 (96.7%)	p < 0.001
	Yes	10 (15.2%)	8 (3.3%)	

The logistic regression model revealed that, in comparison to the control group, psoriasis patients were significantly more likely to have migraine (OR = 2.789; 95% CI 1.722‒4.518, p < 0.001). The risk of migraine substantially increased among psoriasis patients with increasing disease severity (OR = 2.062, 95% CI 1.173‒3.625, p = 0.012; OR = 3.248; 95% CI 1.649‒6.397, p = 0.001; and OR = 4.586; 95% CI 2.360‒8.912, p < 0.001 for mild, moderate, and severe, respectively), and with the presence of PsA (OR = 2.438, 95% CI 1.488‒3.996, p < 0.001 and OR = 12.930, 95% CI 4.567‒36.607, p < 0.001 for patients with and without PsA, respectively) (Table 4).

**Table 4:** The results of regression model to calculate the migraine odds ratio for patients with psoriasis compared to the control group.

Model	B	S.E.	Sig.	Exp(B)	95% CI	
					Low	High
**General**						
Gender						
Female	Refrence					
Male	−0.395	0.237	0.095	0.674	0.423	1.072
Age	0.003	0.009	0.704	1.003	0.986	1.021
Weight						
Normal	Refrence					
Overweight	0.180	0.269	0.504	1.197	0.706	2.030
Obese	0.470	0.290	0.106	1.599	0.905	2.826
Group						
Control	Refrence					
Psoriasis	1.026	0.246	p < 0.001	2.789	1.722	4.518
**According to disease severity**						
Gender						
Female	Refrence					
Male	−0.403	0.239	0.091	0.668	0.419	1.067
Age	0.002	0.009	0.844	1.002	0.985	1.019
Weight						
Normal	Refrence					
Overweight	0.185	0.271	0.495	1.203	0.707	2.048
Obese	0.456	0.293	0.120	1.577	0.888	2.801
Group						
Control	Refrence					
Mild psoriasis	0.723	0.288	0.012	2.062	1.173	3.625
Moderate psoriasis	1.178	0.346	0.001	3.248	1.649	6.397
Severe psoriasis	1.523	0.339	p < 0.001	4.586	2.360	8.912
**According to PsA existence**						
Gender						
Female	Refrence					
Male	−0.351	0.240	0.144	0.704	0.440	1.127
Age	−0.003	0.009	0.776	0.997	0.980	1.015
Weight						
Normal	Refrence					
Overweight	0.197	0.273	0.472	1.217	0.713	2.079
Obese	0.475	0.295	0.108	1.607	0.902	2.866
Group						
Control	Refrence					
Psoriasis without PsA	0.891	0.252	p < 0.001	2.438	1.488	3.996
Psoriasis with PsA	2.560	0.531	p < 0.001	12.930	4.567	36.607

## Discussion

The present results demonstrated a significantly higher migraine frequency in the psoriasis group as compared to the control group (p < 0.001). Furthermore, the logistic regression analysis results showed that the migraine odds ratio in psoriasis patients was considerably higher than the control group (OR = 2.789, p < 0.001). In general, few studies have addressed the association between migraine and psoriasis. For instance, a retrospective population-based cohort study by Min et al. in South Korea showed that the hazard ratio of migraine occurrence in psoriatic patients was notably higher than in the non-psoriatic control group (HR = 1.16, p < 0.05).^18^ The results of another retrospective cohort study in Denmark revealed a significantly higher incidence rate ratio of migraine in psoriatic patients than the control group without a history of psoriasis.^19^ The results of this study corroborate the findings of these two cohort studies. Moreover, a cross-sectional US study evaluated the relationship between migraine and psoriasis based on the medical history of participants in the Health and Nutrition National Survey. It showed that migraine risk in people with psoriasis history was remarkably higher than those without psoriasis history (OR = 3.97, p < 0.003),^21^ which is in line with the present findings. Similarly, the results of a cross-sectional study in Brazil examining the comorbidities in psoriatic patients based on the medical history of participants in the National Survey of Health and Well-being demonstrated that the migraine frequency in psoriatic patients was significantly higher than non-psoriatic subjects (40% vs. 20%, p < 0.05)^20^ which is in agreement with this result. Additionally, the results of a recent meta-analysis also indicated the increased risk of migraine in psoriatic patients compared to the non-psoriatic control group (OR = 1.64, 95% CI 1.28‒2.11).^31^

Conversely, the study conducted by Yang et al. in Taiwan^22^ found no significant relationship between migraine and psoriasis (OR = 1.19, p = 0.409), which contrasts with the present findings and the above studies. However, their study was different from ours in that they used the patients' medical history records from a nationally representative dataset in Taiwan, and the researchers did not directly evaluate the patients. In a previous study in Iran, the migraine frequency in psoriasis patients in Semnan was 20.8%,^24^ which is highly consistent with the present finding (21.2%). Contrarily, the migraine frequency was reported at 47% in an Italian study which was two times the result of this study. However, none of these two studies has examined the migraine association with psoriasis.

Based on the present results, the frequency of migraine with aura, compared to migraine without aura, was higher in psoriatic patients with migraine (53% vs. 47%), while the reverse was noted in the control group with migraine (22.2% vs. 77.8%). The comparative analysis proved a significant difference between the two groups regarding the frequency distribution of migraine type so that the frequency of migraine with aura in the psoriasis group was significantly higher than the control group (p = 0.007). In this regard, the present finding agrees with the results of the study by Capo et al.,^23^ which showed that the frequency of migraine with aura in psoriatic patients was significantly higher than in migraine patients without psoriasis (62.5% vs. 17% and p < 0.001). In the study by Taheri et al.,^24^ the predominant migraine type in psoriatic patients was migraine with aura (60%), which is in line with the present finding. However, they didn't examine the relationship between migraine type and psoriasis due to the lack of a control group. This finding is of particular significance and interest in that migraine with aura is shown to be strongly associated with a higher risk of stroke,^32^ atrial fibrillation,^33^ cardiovascular diseases,^34^ and all-cause mortality.^35^

The present study showed that the migraine headache index in psoriatic patients with migraine was significantly higher than in non-psoriatic patients with migraine (p = 0.015). Other studies in this area have not reported the migraine headache index. However, in the study by Capo et al.^23^ psoriatic patients with migraine with aura experienced a significantly more number of headache attacks than non-psoriatic patients having migraine with aura (p < 0.001). However, the authors found no significant relationship between the number of attacks and migraine type.

The current study results indicate no relationship between migraine and psoriasis duration and affected areas. To the best of our knowledge, this relationship has not been studied in the literature so far.

This study displayed a significant direct relationship between psoriasis severity and migraine. The migraine odds ratio increased compared to the control group as the psoriasis severity progressed from mild to moderate to severe (2.1, 3.2, 4.6 respectively; p < 0.05). Egeberg et al.^19^ presented that the migraine incidence rate ratio elevated with psoriasis severity (1.37 for mild and 1.55 for severe psoriasis), and Taheri et al.^24^ showed that the psoriasis severity in migraine patients was significantly higher than patients without migraine. The results of both studies are similar to the present findings. However, in contrast to this finding, Capo et al.^23^ suggested that the psoriasis severity in psoriatic patients without migraine was significantly higher than the psoriasis with migraine subgroup.

The present results also revealed a significant relationship between PsA and migraine. When the authors compared the psoriatic patients with the control group regarding the existence of PsA, the migraine odds ratio increased remarkably in the psoriasis subgroup with PsA (OR = 12.9 in psoriasis with PsA and OR = 2.4 in psoriasis without PsA). These results are in agreement with the study by Egeberg et al.,^19^ which showed that the migraine incidence rate ratio was higher in psoriatic patients with PsA than without PsA (IRR = 9.3 for patients with PsA and IRR = 5.3‒6.4 for patients without PsA).

A paucity of information exists regarding the possible common pathophysiological pathways between migraine and psoriasis. However, some hypotheses have been proposed to explain the underlying mechanism of their relationship. Given the role of inflammation and endothelial dysfunction in migraine pathogenicity,^36^, ^37^ it has been postulated that inflammation-induced endothelial dysfunction in psoriatic patients (via TNF-α, IL-12, and IL-23 axis) may be a possible mechanism linking psoriasis with migraine.^18^ Psoriasis-related pro-inflammatory cytokines and their effects on vasospasm, meningeal inflammation, and sensitization of the pain conduction pathways might influence the increased prevalence of migraine in psoriatic patients.^24^ A major role has been proposed for Calcitonin Gene-Related Peptide (CGRP) in psoriasis pathogenesis.^12^, ^13^, ^38^ Furthermore, CGRP is a well-recognized trigger of migraine.^39^ Thus, neuropeptides are thought to be a potential risk factor for migraine in psoriatic patients.^18^ In the present study, the highest odds ratio of migraine was related to psoriasis patients with PsA. Provided that psoriasis is a chronic inflammatory disease, and that chronic systemic inflammation is more prominent in cases with PsA,^40^ this finding of the present study somehow supports the role of chronic systemic inflammation as a common pathogenic mechanism between psoriasis and migraine. In this regard, the results of another study revealed that most psoriatic patients clinically diagnosed with migraine were suffering from PsA.^23^

One limitation of the present study was that other confounding factors affecting the relationship between migraine and psoriasis were not addressed, including lifestyle, dietary habits, socioeconomic status, and exercise activity. Additionally, due to the observational nature of this study, the authors cannot determine a definite cause-and-effect relationship between migraine and psoriasis. Finally, it should be noted that this study was conducted in Ardabil (Iran), and caution should be made when generalizing the results to other ethnicities because it has been shown that migraine prevalence may vary between different ethnicities.

## Conclusion

In conclusion, the current study suggests that in comparison with the non-psoriatic control group, patients with psoriasis are significantly more susceptible to migraine, particularly migraine with aura; the migraine headache index is considerably higher in psoriasis patients with migraine; the migraine odds ratio is remarkably higher in psoriasis patients with more severe disease and with PsA.

## Financial support

The authors declare that they have no known competing financial interests or personal relationships that could have appeared to influence the work reported in this paper. This research did not receive any specific grant from funding agencies in the public, commercial, or not-for-profit sectors.

## Authors’ contributions

Sarkhani Mohammad participated in the study concept and design, data collection, analysis and interpretation, and writing of the manuscript.

Fattahzadeh-Ardalani Ghasem participated in the data collection and final approval of the final version of the manuscript.

Rostami Mogaddam Majid participated in the data collection and final approval of the final version of the manuscript.

Fouladi Nasrin participated in the statistical analysis.

## Conflict of interest

None declared.
